# Supplementary material for: An exploratory study of outpatient medication knowledge and satisfaction with medication counselling at selected hospital pharmacies in Northwestern Nigeria
Source: PLoS One. 2022 Apr 8;17(4):e0266723. doi: 10.1371/journal.pone.0266723 (PMC8992974; doi:10.1371/journal.pone.0266723)
Supplement: Attachment — Submitted filename: Responses to review comments - PLoS one.docx [file pone.0266723.s002.docx]

**Response to Reviewers comment:**

*Dear Reviewer.*

*Thank you so much for taking your time to review our paper and offer constructive input. Please find below our response to the comment raised during the second review. Thank you again.*

**Reviewers’ comment:** One thing (in results), the authors didn't address a summary measure of overall medication knowledge for a reason of wide range medications they assessed. However, this is not about picking a random number/figure as they claimed. They are supposed to evaluate correctness of outpatients for each item testing knowledge of users/guardians about the medicines they received. In this regard, correct answers of the users for all questions assessing knowledge can be evaluated for a summary measure description of the knowledge. Otherwise, it is doubtful about their systematic assessment of the clients' knowledge at exit from drug outlets.

**Action(s) taken, with reason(s) where relevant:** This is noted. A summary measure of overall medication knowledge has now been included in the manuscript (see lines 263 to 264). A few lines have also now been added to the methods section (lines 185-191) describing how the summary measure was calculated.
